# Effects of Bisphenol A on Oxidative Stress in the Rat Brain

**DOI:** 10.3390/antiox9030240

**Published:** 2020-03-16

**Authors:** Keiko Kobayashi, Yanchen Liu, Hiroshi Ichikawa, Shigekazu Takemura, Yukiko Minamiyama

**Affiliations:** 1 Food Hygiene and Environmental Health Division of Applied Life Science, Graduate School of Life and Environmental Sciences, Kyoto Prefectural University, Sakyo-ku, Kyoto 606-8522, Japan; k-kobayashi@kpu.ac.jp; 2 Department of Medical System Protective Health and Medicine Laboratory, Graduate School of Life and Medical Sciences Doshisha University, Kyotanabe 610-0394, Japan; chrisliu93@gmail.com (Y.L.); hichikaw@mail.doshisha.ac.jp (H.I.); 3 Department of Hepato-Biliary-Pancreatic Surgery, Graduate School of Medicine, Osaka City University, Abeno-ku, Osaka 545-8585, Japan; takemura@med.osaka-cu.ac.jp

**Keywords:** bisphenol A, dementia, oxidative stress, tau

## Abstract

We investigated the effect of bisphenol A (BPA) on oxidative stress and tau-related proteins in adult rat brains. BPA (10 mg/L) was administered to rats for eight weeks through their drinking water. The reactive oxygen species (ROS) scavenging capacity for hydroxyl radicals in the plasma was reduced after two weeks. In the hippocampus, four and eight weeks of BPA increased the ratio of oxidized DJ-1/DJ-1 (PARK7). The ratio of phosphorylated-GSK3β/GSK3β and phosphorylated-AKT/AKT increased after one week of BPA treatment. The ratio of phosphorylated JNK/JNK and phosphorylated-ERK/ERK increased after eight weeks of BPA; the elevation could be related to tau phosphorylation. Protein phosphatase 2A (PP2A) in the hippocampus decreased after eight weeks of BPA treatment. At that time, SOD1 was significantly induced, but no changes in SOD2 expression were apparent in the hippocampus. Furthermore, the ratio of phosphorylated-tau (PHF-1, Ser396/ Ser404) to total tau level did not change. However, PHF-1 or other sites of tau could be phosphorylated after eight weeks in the hippocampi of rats. BPA induced systemic oxidative stress and could change ROS-induced signaling pathways in the brain. These results suggest that mitochondrial dysfunction possibly is not responsible for oxidative stress and neurodegeneration due to low doses of BPA.

## 1. Introduction

The worldwide production of bisphenol A (BPA) is higher than that of any other chemical. BPA is used on the production of polycarbonate plastics and epoxy resins used in many consumer materials, including toys, water pipes, containers, medical equipment, and consumer electronics [1]. BPA can leach from food and beverage containers [1,2]. When consumed, BPA is quickly absorbed in humans and broken down into its metabolites [3], and growing evidence suggests that free BPA can, then, circulate throughout the body [4]. The discovery of BPA in children’s urine prompted banning the use of this chemical in children’s products. However, low-dose BPA remains in food-contacting materials such as canned coating agent.

Human exposure to BPA through inhalation, ingestion, or absorption results in circulating levels of BPA in the range of 10 to 100 nM [5]. Free circulating BPA that is structurally similar to diethylstilbestrol (DES) is an endocrine disruptor; however, it is substantially weaker than DES or estradiol [6]. Furthermore, BPA can stimulate cellular responses at very low concentrations through various pathways. The molecular mechanism most frequently implicated in the detrimental action of BPA is its binding to and activation of estrogen receptors α and β (ERα and ERβ, respectively) [5]. In addition, BPA is a potent activator of nonclassical estrogen receptors, such as the G-protein coupled receptors (GPCRs) and estrogen-related receptor γ (ERRγ) [7]. There is growing evidence that BPA alters reproduction, development, metabolism, immune response, and neurobehavior through its estrogenic action [8]. Moreover, research has shown that exposure to low doses of BPA induces oxidative stress in the liver, kidney, and reproductive organs [8].

BPA increases the levels of reactive oxygen species (ROS) via enzymatic (H_2_O_2_/peroxidase and NADPH/CYP450) and non-enzymatic (peroxynitrite/CO_2_ and OCl^-^/HOCl) formation of phenoxyl radicals, which react with NADPH or glutathione (GSH) to produce a variety of radical species, including superoxides, peroxides, and hydroxyl radicals [9]. Various doses of BPA cause DNA damage, cytotoxicity, and mitochondrial dysfunction both in vitro and in vivo [8]. Previously, we have reported that BPA exposure induces sperm dysfunction via mitochondrial ROS generation in rats [10]; however, its effect on the brain remains unclear. Furthermore, it has been reported that pubertal exposure to BPA affects social play and sociability in adolescent male mice through changes in the androgen and dopamine pathways [11]. Thus, BPA can affect the adult brain.

This study investigated the BPA-induced oxidative stress and metabolic effects on tau-related proteins in BPA-treated rat brains.

## 2. Materials and Methods

### 2.1. Animals

Twelve-week-old male Wistar rats were purchased from CLEA Japan, Osaka. Rats were kept on a 12 h light-dark cycle with free access to food and water. Rats (*n* = 3 to 6) were separated into two groups and fed with either water (control) or 10 mg/L BPA for 1, 2, 4, or 8 weeks. Rats were anesthetized using Sevoflurane (MARUISHI Pharmaceutical, Osaka, Japan) before the intraperitoneal administration of 5 g/kg of 25% ethyl carbamate (WAKO, Osaka, Japan) and were sacrificed at the age of 22 weeks. After dissection, blood was collected from the inferior vena cava, and brain tissues were collected after saline perfusion. The hippocampus and plasma samples were immediately stored at −80 °C until analyses were performed. This study was conducted in compliance with the guidance for the Use of Laboratory Animals of Doshisha University (No. A18065 & A17061).

### 2.2. Measurement of Plasma Free Radical Species Scavenging Capacity

The plasma scavenging capacity for different free radical species was measured according to the method reported by Oowada et al. [12]. A spin trapping agent, 5-(2,2-dimethyl-1,3-propoxy cyclophosphory)-5-methyl-1-pyrroline N-oxide (CYPMPO, Radical Research Inc, Tokyo, Japan), was used to measure the scavenging capacity of each free radical with an X-band Microwave Unit ESR (RE SERIES, JEOL, Tokyo, Japan). WIN-RAD (Radical Research Inc, Version 1.30, Tokyo, Japan) was used for data analysis. Briefly, we prepared a solution containing 20 µL of 1:5 diluted plasma samples, 20 µL of 100 mM CYPMPO, 20 µL of 10 mM dimethylene triamine pentaacetic acid (DTPA, WAKO, Osaka, Japan), 20 µL of distilled water, and 20 µL of 100 mM H_2_O_2_ for hydroxy radical generation. This was exposed to UV light (SUPERCURE-203, SAN-EI ELECTRIC, Osaka, Japan) for 5 s (total reflection mirror, hot-cut filter). CYPMPO was used as a spin trapping reagent to capture any radicals produced in the solution. The height of the fifth signal of each sample was recorded. The fifth signal of control was set to 100% and compared with each sample to obtain a relative ratio. The standard calibration curve was determined using glutathione disulfide (GSSG, WAKO, Osaka, Japan). The relative ratios of the control and each concentration were set as I0 and I, respectively, when GSSG was added to each sample. In the graph created from these results, the y-axis and x-axis represented I0/I-1 and the concentration of GSSG (mM), respectively. For other free radicals, such as superoxide (O_2_•^−^), alkoxy radical (RO•), methyl radical (CH_3_•), alkylperoxyl radical (ROO•), and singlet oxygen (^1^O_2_), each suitable reaction mixture was used and measured for each condition. These standard curves are shown in Supplementary Figure S1. Typical spectrometer settings were as follows: field modulation width, 0.1 mT; microwave power, 6 mW; field scan width and rate, ±7.5 mT/2 min; and time constant, 0.1 s.

### 2.3. Western Blotting

A radioimmunoprecipitation assay buffer was added to hippocampal samples with a volume nine-fold the weight of the tissue and homogenized over ice (NS-360D, MICROTECH, PA, USA) at a speed of 20,000 rpm for 30 s. Samples were then centrifuged (CT15RE, HITACHI, Tokyo, Japan) at 4 °C and 10,000× *g* for 10 min. The supernatant was collected and used for further analyses. Total protein concentration was measured using a BCA Protein Assay Kit (THERMO, MA, USA).

Each sample (10 µL) was loaded onto an SDS-PAGE gel to obtain a final protein amount of 0.2 to 10 µg depending on the antibodies used. The gel was run at 50 or 30 mA for four or two gels, respectively (BIO-RAD, CA, USA), for approximately 70 min. PM2500 (SMOBIO, Taiwan, Hsinchu) or dual color standards (BIO-RAD, CA, USA) protein marker was used depending on the antibody used. The PVDF membrane (MILLIPORE, MA, USA) was activated in 100% methanol for 30 s. The membrane was then washed twice with distilled water, followed by incubation in the blotting buffer for at least 30 min. The filter paper was also soaked in the blotting buffer before the end of the gel run. Protein transfer was performed using a semi-dry blotting machine (ATTO, NY, USA). Membrane and gel were sandwiched between two layers of three filter papers and run at constant current (140 mA) for 70 min. After protein transfer, the membrane was blocked with 5% skim milk, 4% blocking-s or blocking one-P according to the antibody type at room temperature for one hour. Next, membranes were incubated with primary antibodies at 4 °C overnight. Antibodies were dissolved in Canget Signal Solution 1 (TOYOBO, Osaka, Japan) and diluted to appropriate concentrations. Primary antibodies included the following: DJ1 (1:2000; Abcam, U.K. Cambridge, Lot GR37461-12); oxidized DJ-1 (oxDJ-1) (1:2000; Bio-Rad, CA, USA, HCA024), Akt (1:2000; Cell Signalling Technology, MA, USA, Lot 9), phospho-Akt (1:1000; CST, MA, USA, Lot 14), phosphorylated Tau (paired helical filaments, PHF1) (1:2000; gifted from Prof. Miyasaka at Doshisha University), total tau (1:2000; BD Pharmingen, CA, USA, Lot 3102902), extracellular signal-regulated kinase (ERK1/2) (1:1000; CST, MA, USA, Lot 16), p-ERK (threonine-202 and tyrosine-204 residues) (1:2000; CST, MA, USA, Lot 19), glycogen synthase kinase 3 (GSK3β) (1:5000; CST, MA, USA, Lot 3), p-GSK3β Ser9 (1:5000; CST, MA, USA, Lot 15), c-Jun N-terminal kinase (JNK) (1:1000; CST, MA, USA, Lot 17), p-JNK (threonine-183 and tyrosine-185 residues) (1:1000; CST, MA, USA, Lot 10), amyloid precursor protein (APP) (1:2000; CST, MA, USA, Lot 2), PP2A (1:5000; Abcam, U.K. Cambridge, Lot GR96004-27), SOD1 (1:50,000; Novus Biologicals, CO, USA, Lot YED11501R), SOD2 (1:5000; Abcam, U.K. Cambridge, Lot 645437), and β-actin (1:10000; CST, MA, USA, Lot 7). After incubation in the primary antibodies overnight, membranes were washed with TBS-T for 3 × 5 min. Secondary antibody incubation was performed with polyclonal goat anti-rabbit immunoglobulin/horseradish peroxidase (HRP) (1:20000; DAKO, USA) or polyclonal goat anti-mouse immunoglobulin/HRP (1:20000; DAKO, Santa Crala, CA, USA) at room temperature for 60 min. Membranes were subsequently washed with TBS-T for 3 × 5 min, and immobilon western chemiluminescent HRP (MILLIPORE, MA, USA) was then added to the membranes for 2 min to obtain a color reaction. Bands were visualized using a LAS-4000 (FUJI FILM, Tokyo, Japan). GAPDH or β-actin was used as a loading control.

### 2.4. Statistical Analysis

Data analysis was performed using IBM SPSS v24 (IBM, Armonk NY, USA). One-way ANOVA was used to compare between 2 groups to identify specific differences from the ANOVA result. Results were considered significant when *p* was <0.05.

## 3. Results

### 3.1. Free Radical Scavenging Capacity in BPA-Treated Plasma Samples

**Figure 1:**
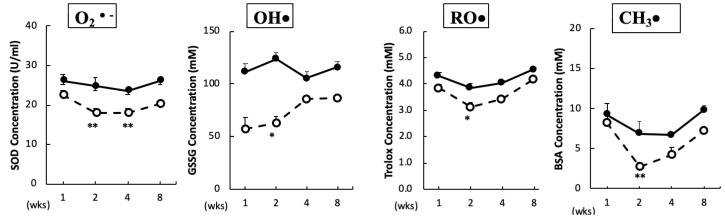
Free radical scavenging capacity in plasma. Animals were treated as described in the Methods Section. Plasma samples in each group were measured at the indicated times. Values represent mean ± SD (*n* = 3 to 6). Solid line, control and dotted line, bisphenol A (BPA). * *p* < 0.05, ** *p* < 0.01 vs. control group (a two-way ANOVA).

The O_2_•^–^ scavenging capacity in plasma was significantly reduced after two and four weeks of BPA treatment. In addition, the scavenging capacities for OH•, RO•, and CH_3_• in plasma were significantly reduced after two weeks of BPA treatment as compared with the control treatment (Figure 1). No significant changes were observed in the ROO• and ^1^O_2_ scavenging capacities between the groups (data not shown).

### 3.2. Effect of BPA on Oxidative Stress in the Brain

**Figure 2:**
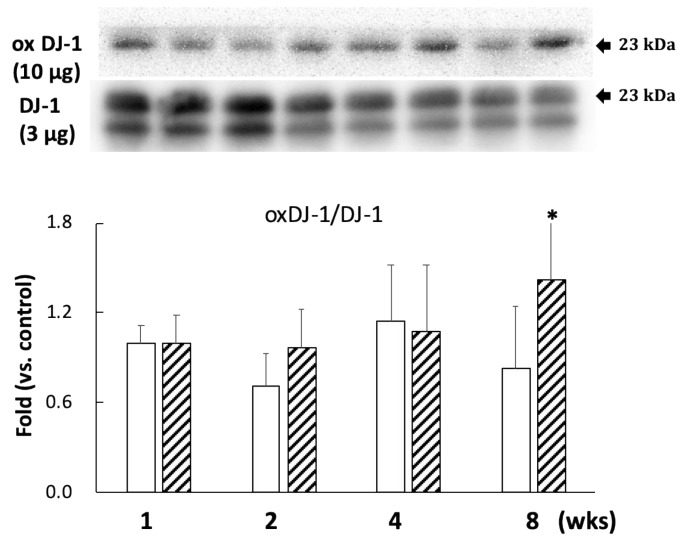
Effect of BPA treatment on the ratio of oxDJ-1 (10 µg/lane)/DJ-1 (3 µg/lane). Open column, control and gray column, BPA. Values represent mean ± SD (*n* = 3 to 6); * *p* < 0.05 vs. control group.

**Figure 3:**
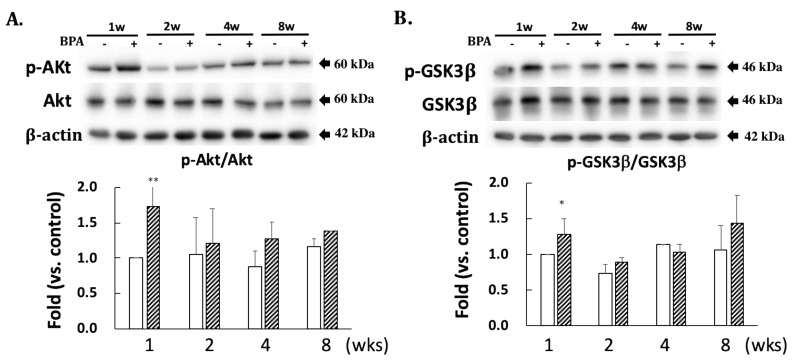
Effect of BPA treatment on phosphorylated Akt/total Akt (**A**); and phosphorylated GSK3β/total GSK3β (**B**). Open column, control and gray column, BPA. Values represent mean ± SD (*n* = 3 to 6), * *p* < 0.05 and ** *p* < 0.01 vs. control group.

A comparison with DJ-1 levels in control specimens showed that those in the BPA-treated hippocampus samples did not show an effect during this study (Figure 2). Cysteine-106 residue in DJ-1 is essential for its protective function of oxidation [13]. The oxidation of DJ-1 (oxDJ-1) significantly increased in BPA-treated hippocampus samples after eight weeks (Figure 2). Akt is another cytoprotective protein. The PI3K/Akt survival signaling pathway is regulated by oxidative stress, and Akt exerts its cytoprotective action through phosphorylation at the serine-473 residue [14]. While no significant changes were observed in the total Akt level in any of the groups (Figure 3A), the BPA-treated group featured a significant increase in p-Akt after one week (Figure 3A), which downregulates GSK3β activity by phosphorylating it at Ser-9 [15]. Consistent with an increase of p-Akt, BPA increased phosphorylated-GSK3β (Figure 3B).

### 3.3. The Effect of BPA on MAP Kinases

**Figure 4:**
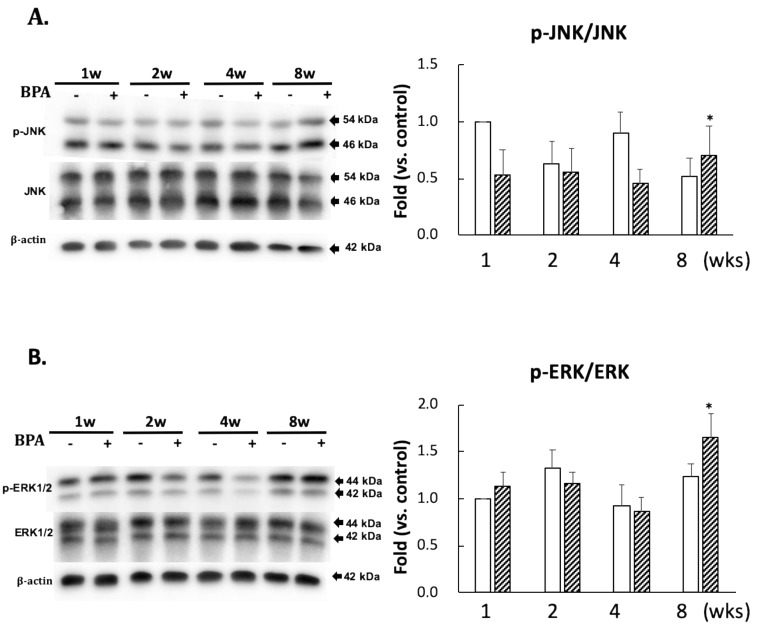
Effect of BPA treatment on phosphorylated JNK/total JNK and (**A**); and phosphorylated ERK/total ERK and (**B**). Open column, control and gray column, BPA. Values represent mean ± SD (*n* = 3 to 6), * *p* < 0.05 vs. each control group.

No significant differences in the levels of p-JNK/JNK and p-ERK/ERK ratios between the two groups were observed until eight weeks of administration (Figure 4). However, there was a significant increase in their ratios after eight weeks of BPA treatment as compared with the control treatment (Figure 4).

### 3.4. The Effect of BPA on Amyloid Precursor Protein (APP) and Tau Protein

**Figure 5:**
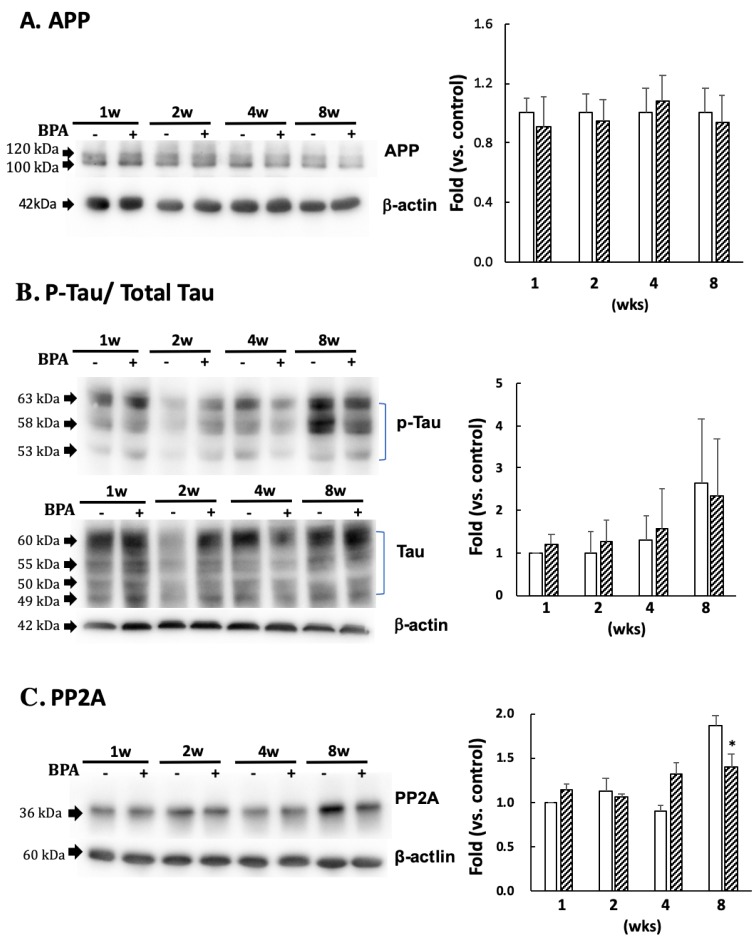
Effect of BPA on APP (**A**); phosphorylated tau protein (PHF1 epitopes)/total Tau (**B**); and PP2A (**C**) levels. Open column, control and gray column, BPA. Values are mean ± SD (*n* = 3 to 6). * *p* < 0.05 as compared with each control group.

There was no significant difference in APP (Figure 5A) or p-tau (PHF1 epitopes)/total tau (Figure 5B) between the groups. PP2A decreased after eight weeks of BPA treatment as compared with the control; no differences were observed at any other time (Figure 5C).

### 3.5. The Effect of BPA on Superoxide Dismutase

**Figure 6:**
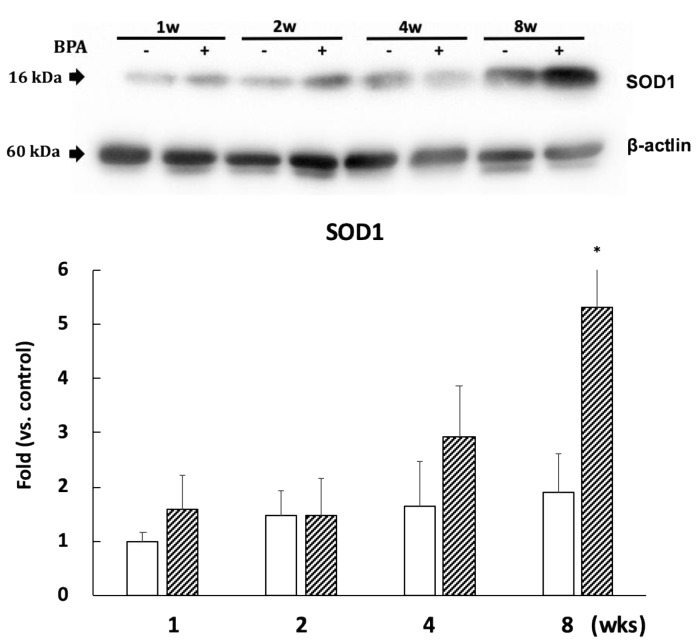
Effect of BPA on SOD1 levels. Open column, control and gray column, BPA. Values are mean ± SD (*n* = 3 to 6). * *p* < 0.05 as compared with each control group.

SOD1 expression significantly increased after eight weeks of BPA treatment as compared with controls (Figure 6). However, the levels of SOD2 expression did not differ between the groups (Supplementary Figure S2).

## 4. Discussion

This study revealed that BPA induced oxidative stress and changed ROS-induced signal pathways associated with tau-related proteins in the plasma and brain of rats.

A previous epidemiological study found that long-term low-dose BPA exposure could affect reproductive, developmental, and metabolic diseases by causing oxidative stress [16]. We found that BPA administration resulted in a reduction in ROS scavenging capacity in plasma, indicating that BPA can decrease the antioxidative capacity (Figure 1). These results are consistent with a previous report showing that a reduction in antioxidant activity correlates with dose-dependent BPA-induced ROS activity [8]. Higher doses of BPA (for example, 30 mg/kg/day for six weeks, which is 30,000 times concentration of the dose used in our study) have been shown to induce oxidative damage in the liver and heart of rats [17]. Furthermore, administration of 50 µg/kg/day of BPA for 10 weeks to mice has been shown to induce apoptosis, oxidative stress, and inflammatory response in the colon and liver via the mitochondrial pathway [18]. However, we did not observe a decrease of SOD2 or an increase of caspase 9 in the BPA-treated hippocampus (see Supplementary Figure S2 for SOD2 data, caspase 9 data not shown). Therefore, the decrease of plasma antioxidant capacity can be an additive effect due to damage to the whole body caused by BPA. In the hippocampus, we confirmed that BPA-induced free radicals triggered oxidative stress in the brain and significantly increased oxDJ-1/DJ-1 ratios after eight weeks of treatment (Figure 2). DJ-1 is a multifunctional redox-sensitive protein that is associated with the oxidative stress cell death cascade [19]. DJ-1 provides neuroprotection in multiple pathways, most importantly by reducing mitochondrial oxidative stress [20]. Our results could be due to an imbalance in the redox reaction that exceeded cellular recovery capacity. Furthermore, hippocampal levels of p-Akt significantly increased after one week of treatment (Figure 3A), which further confirmed that BPA induced oxidative stress in the brains of these rats. More experiments are required to confirm the reliability of BPA-induced oxidative stress, such as examining other markers of oxidative stress or rescue studies with antioxidants. We previously reported that BPA-induced oxidative stress in rat sperm is protected by the administration of the antioxidant N-acetylcysteine [10]; antioxidants can provide the same protection in the brain.

Furthermore, p-Akt phosphorylates GSK3β to inhibit its activity. We found significant increases in p-Akt, and GSK3β phosphorylated at the serine 9 residue one week after BPA treatment (Figure 3A,B). This suggests that the increase in p-Akt could be a transient adaptation to inactivate GSK3β, resulting in the inhibition of tau phosphorylation.

The kinase and phosphatase pathways are involved in abnormal tau phosphorylation [21]. Furthermore, many kinases are associated with tau phosphorylation. In this study, we measured the protein expression of JNK, ERK 1/2, and phosphatase PP2A, which are essential for tau dephosphorylation. Many observations in vitro suggest that PP2A plays a major role in the downregulation of the ERK pathway. Kins et al. [22] reported that chronic inhibition of PP2A in mice causes the activation of ERK and JNK. Our data indicated that the decreased PP2A (Figure 5C) was consistent with phosphorylation of ERK and JNK (Figure 4) in BPA-treated hippocampus at eight weeks. AT8 (Ser202/Thr205) and epitope Ser422 are excellent substrates of ERK and JNK in in vitro studies [23,24]; however, AT8 was not detected in the hippocampus (data not shown). Tau protein has many sites of phosphorylation; therefore, other than that of p-tau at the serine 404 and 396 residues (PHF1), AT8 can be phosphorylated.

It has been reported that p-ERK of the MAPK family has both detrimental (promoting inflammation and oxidative stress) and beneficial (produced by estrogen, and preconditioning, etc.) effects for the brain [25]. Our data indicated that the increase of oxDJ-1 and the activation of MAPK family occurred at the same time. Because ROS has been reported to activate the MAPK family [26], ROS could be directly linked to the regulation of ERK or JNK/MAPK.

Finally, other cells or organelles in different parts of the brain can be damaged by ROS and result in neuronal loss. Studies have found that mitochondrial dysfunction and also vascular endothelial damage and the dysfunction of the renin-angiotensin system are also involved in Alzheimer’s disease (AD) development [27]. The induction of SOD1 expression and unchanged expression of SOD2 observed in the hippocampus in this study suggest that mitochondrial dysfunction did not occur. In particular, the induction of SOD1 after eight weeks of BPA treatment could be an adaptation for oxidative stress. Therefore, further investigations are required to determine whether BPA-induced oxidative stress could affect these systems and their components, including microglia, macrophages, and endothelial cells.

## 5. Conclusions

**Figure 7:**
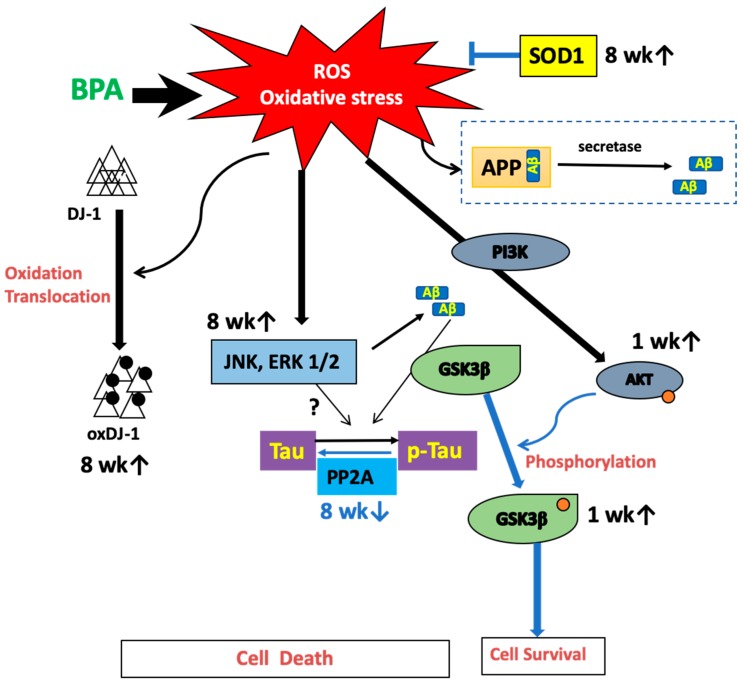
Hypothetical pathways of BPA-induced oxidative stress and tau-related proteins.

Overall, we showed that BPA induced oxidative stress in the plasma and the hippocampus. We have hypothesized a possible mechanism that summarizes our results (Figure 7). However, it should be noted that this study was not performed in a model of AD. It has been reported that subcutaneous injection of 100 µg/kg/day for four weeks in mice induced a significant decrease of insulin sensitivity and hyperactivation of the IR/IRS/AKT/GSK3b axis in the brain [28]. Future investigations should be performed in female rats to assess continuous BPA intake and confirm whether these changes could cause either dementia or memory loss. Moreover, substantial evidence indicates that oxidative stress plays an important role in AD progression [29]. Alzheimer’s disease is a progressive form of dementia. The World Alzheimer Report published in 2015 by Alzheimer’s disease International (ADI) estimates that 46.8 million people worldwide live with dementia and predicts that this number will reach 131.5 million in 2050. An increase in AD-related proteins could result in neuronal damage from a long-term low-dose synergistic accumulation of BPA and exposure to other chemicals, such as food additives.

## Supplementary Materials

The following are available online at https://www.mdpi.com/2076-3921/9/3/240/s1, Figure S1: Standard of free radical scavenging activity on ESR measurement, Figure S2: Representative figure of SOD2 levels of BPA treatment.
