# Supplementary material for: Effects of Bisphenol A on Oxidative Stress in the Rat Brain
Source: Antioxidants (Basel). 2020 Mar 16;9(3):240. doi: 10.3390/antiox9030240 (PMC7139612; doi:10.3390/antiox9030240)
Supplement: Supplementary file 1 [file antioxidants-09-00240-s001.pdf]

Suppl. 1

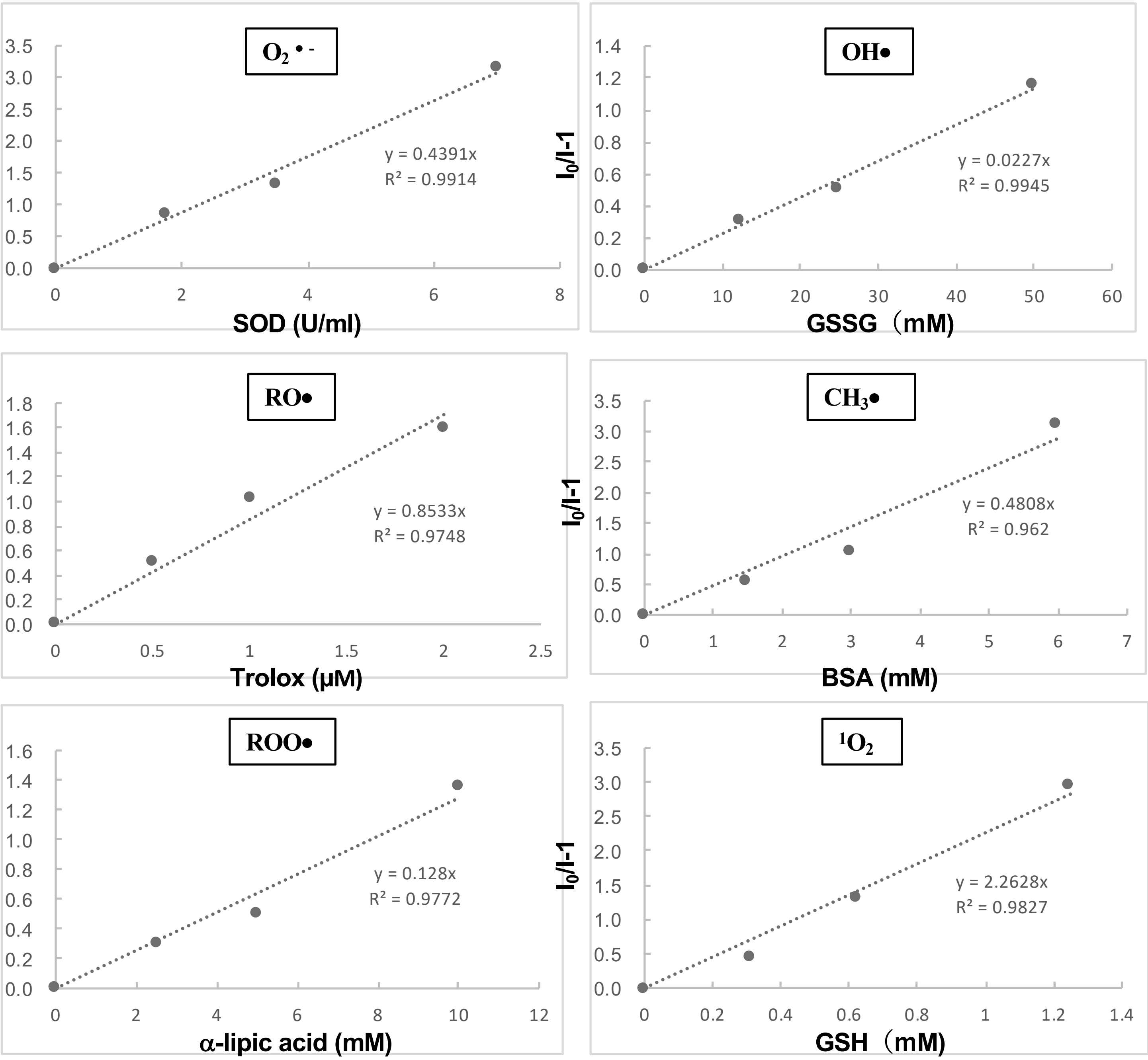

Suppl. 1 Standard of free radical scavenging activity on MULTIS method

Suppl. 2

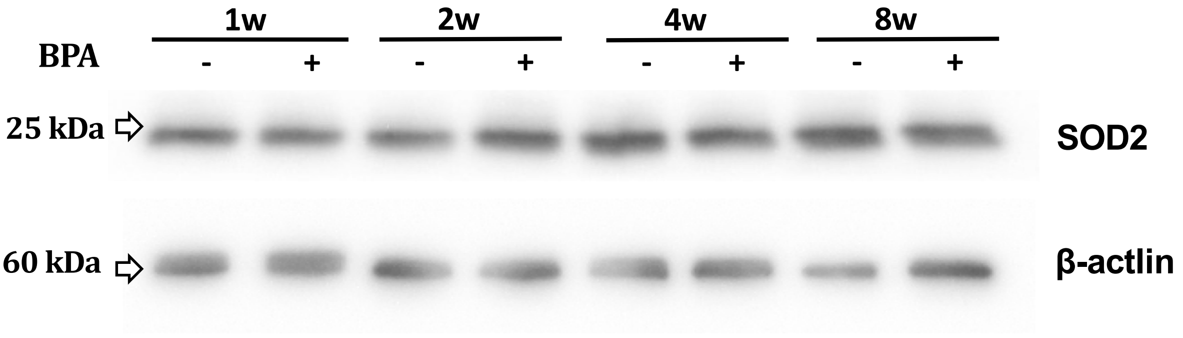

Suppl. 2 Representative figure of SOD2 levels of BPA treatment.
